# Intergenerational ambivalence among families with a migrant background caring for older relatives

**DOI:** 10.1016/j.jmh.2024.100244

**Published:** 2024-07-14

**Authors:** Sunita Shrestha, Alistair Hunter, Jonas Debesay, Sanjana Arora

**Affiliations:** a Department of Nursing and Health Promotion, Faculty of Health Sciences, Oslo Metropolitan University, Norway; b School of Social and Environmental Sustainability, University of Glasgow, Dumfries, UK; c Centre for Intercultural Communication, VID Specialized University, Stavanger, Norway

**Keywords:** Family caregiver, Migrant, Aging, Rotational care, Privacy, Intergenerational ambivalence

## Abstract

**Introduction:**

Europe's population is aging and becoming more ethnically diverse due to migration. The growing number of aging migrants has raised concerns about their future eldercare arrangements and their implications for both families and formal care services. Many older non-European migrants prefer family care over formal, long-term care facilities.

**Objective:**

The objective of this study is to explore the family caregiving arrangements for older Pakistani migrants from the Ahmadiyya community in Norway.

**Methods:**

This exploratory qualitative study recruited 19 women between 25 and 62 years of age who were family caregivers for older adults. Semi-structured individual (18) and group interviews (2) were conducted in Urdu and English.

**Findings:**

Our analysis reveals four main themes: the need to share caring responsibilities with family, balancing personal relations in managing care, lack of privacy while caregiving, and feelings of inadequacy.

**Conclusion:**

While rotational care for older family members offers benefits such as the sharing of responsibilities and more privacy, reliance solely on family care arrangements led to greater ambivalence among family caregivers and provoked doubts about the ability to continue caring for older relatives in the future. Our findings highlight the urgent need to establish modes of collaboration with formal care systems to ensure the support and well-being of both older adults and their family caregivers.

## Highlights

• This is the first study on intergenerational care among a religious minority in Norway.
• Caregiving with multiple roles and privacy concerns led to intergenerational ambivalence.
• A mismatch between care ideals and actual care led to caregiver guilt.
• There is increasing need for male siblings’ involvement in care.
• Some families provide emotional support but are open to formal care for physical tasks.

## 1 Introduction

Europe's population is aging and becoming more ethnically diverse due to migration (WHO, 2018). In Norway, projections indicate a significant increase in older migrants, mainly from non-European countries, with a potential fourfold rise in those over the age of 80 in the next 20 years (Syse and Tønnessen, 2022b). As people age, their healthcare needs increase, and multimorbidity becomes more common (Northwood et al., 2018). Non-European older migrants often report lower self-rated health, well-being, and mental health compared to the majority population (WHO, 2018). Women are particularly vulnerable due to relatively lower income and labor force participation. Thus, the growing number of aging migrants has raised concerns about their future eldercare arrangements and their implications for both families and formal care services (Syse and Tønnessen, 2022a).

The welfare state in Norway ensures universal access to health and social care services through municipalities, although increased deinstitutionalization has shifted care responsibilities to families (Vangen and Herlofson, 2023). Many older non-European migrants prefer family care over formal, long-term care facilities (Sagbakken et al., 2018). Their lower use of formal services may be due to lower socio-economic status and barriers related to limited information availability, language skills, and culturally insensitive services (Debesay et al., 2022; Shrestha et al., 2023). While family care is often viewed as an individual choice in Norway's comprehensive welfare state (Vangen and Herlofson, 2023), cultural norms and filial and moral obligations stemming from collectivist traditions heavily influence family caregiving among migrants (Albertini et al., 2019; Horn, 2023). Non-compliance with these norms can lead to feelings of guilt and shame as well as social exclusion; therefore, migrant families tend to prioritize others’ needs over their own (Tavernier and Draulans, 2018).

Family caregiving encompasses forms of support ranging from completing daily tasks to navigating complex healthcare systems (Schulz et al., 2020). It can foster personal growth, satisfaction, and closer relationships with care recipients (Zarzycki et al., 2023). However, it often takes a toll on caregivers that may lead to poor health outcomes (Schulz et al., 2020; Stenberg and Hjelm, 2023) and ambivalence and insecurity in intergenerational family relations, which can reduce their psychological well-being and quality of life (Lüscher and Hoff, 2013; Yao, 2023). Gender disparities are more pronounced in caregiving duties, particularly among migrant women (Khan, 2023; Næss and Vabø, 2014), and female caregivers are likely to experience ambivalence differently than men due to gendered expectations around care (Connidis and McMullin, 2002; Willson et al., 2003). The high care burden among women often overlaps with childrearing and employment (Syse and Tønnessen, 2022b), which can cause sibling conflicts (Shrestha et al., 2023). Nevertheless, most adult children feel responsible for providing care for their migrant parents (Sun, 2023).

Several studies in Norway have explored caregiving for older migrants, predominantly in relation to their access to formal healthcare services (Arora et al., 2020; Næss and Vabø, 2014; Sagbakken et al., 2018) or in the context of people with dementia (Gulestø et al., 2023; Lillekroken et al., 2023; Sagbakken et al., 2018). Yet, it is crucial to consider older adults who live at home without specific diagnoses, and additional research is needed to understand how care is managed within families with a migrant background (de Valk and Bordone, 2019; Horn, 2023). Exploring the increasing, overlapping roles of family caregivers and the quality of intra-generational relations is important for shaping inter-generational care arrangements for older migrant parents. To date, no study has investigated caregiving arrangements among a minority Muslim group in Norway (e.g., the Ahmadiyya community). By examining the practical aspects of caregiving for older Pakistani migrants within the Ahmadiyya community in Norway, this study aims to deepen our understanding of family caregiving arrangements for older migrants.

## 2 Theoretical framework

To analyze family caregiving arrangements for older migrants, we use the concept of intergenerational ambivalence (IGA). The intergenerational family in post-modern society is complex due to population aging, increased life expectancy, a greater need for informal care, and more diverse family structures (Connidis, 2015; Lüscher and Hoff, 2013; Yao, 2023). The notion of IGA aims to capture and describe this complexity. Additionally, immigration to a new country can often lead to change in circumstances of caregiving and acculturation varies between generations, which further complicates intergenerational relationships (Berry, 2005; Schwartz et al., 2010).

**Fig. 1:**
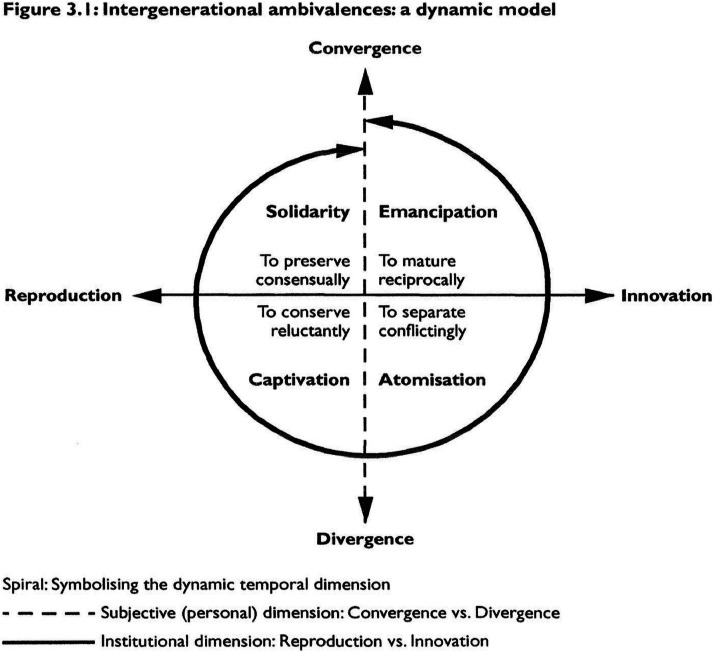
Lüscher's Model of Intergenerational Ambivalence (Derived from (Lüscher and Hoff, 2013)).

Intergenerational ambivalence has two dimensions: psychological ambivalence at the individual level and structural ambivalence at the socio-structural level (Lüscher and Hoff, 2013; Park, 2014). These dimensions intersect to form the four sub-dimensions of solidarity, captivation, atomization, and emancipation, which represent modes of coping with ambivalence in intergenerational relations (Lüscher and Hoff, 2013). The subjective dimension reflects the differentiated development of personalities between adult children and their older parents and is depicted as *Convergence*, indicating love and closeness, and *Divergence*, implying indifference and autonomy. The structural dimension represents the contradictory situation of preserving traditional norms through *Reproduction* or changing them through *Innovation* (Lüscher and Hoff, 2013; Park, 2014) (Fig. 1).

*Solidarity* in coping with IGA emphasizes common feelings and togetherness to avoid ambivalence, with both parents and children being reluctant to change norms. *Captivation* involves a continuous struggle over ambivalence in which one generation, usually parents, imposes their will on others through moral pressure, and ambivalent feelings are not acknowledged directly; thus, care norms are reluctantly maintained. *Atomization* entails disengagement to avoid conflict and ambivalence while *Emancipation* implies an open acknowledgment of ambivalence that allows individuals to accept both conflict and cooperation, thereby fostering closeness and embracing new norms (Lüscher and Hoff, 2013; Park, 2014). Unlike the traditional theoretical approaches to solidarity, which overlook power dynamics, and the conflict approaches, which oversimplify relationships as positive and negative, IGA acknowledges the simultaneous presence of solidarity and conflict within intergenerational dynamics (Connidis, 2015; Lüscher and Pillemer, 1998; Park, 2014). In this study, IGA provides a theoretical lens for understanding the ambivalent experiences of adult children in their daily caregiving for older migrant parents.

## 3 Methods

A qualitative study using semi-structured interviews (John and Creswell, 2023) was employed to explore family caregivers’ experiences of the practical aspects of caregiving for older Pakistani migrants in the Ahmadiyya community in Norway.

### 3.1 Setting

The Pakistani community in Norway is one of the largest and longest-residing non-European migrant groups (Ingebretsen et al., 2015). Members of this community began immigrating in the late 1960s as labor migrants and were later joined by family members (Ingebretsen et al., 2015). Norway's total population of Pakistani immigrants and Norwegian-born children of Pakistani immigrant parents is 42,231 (SSB, 2024). Ahmadis, who are a religious minority in Pakistan, migrated to Norway to escape religious persecution by mainstream Muslims (Jacobsen et al., 2015). The approximately 1200 Ahmadis living in Norway (Høydal et al., 2023) comprise a minority within the larger ethnic group of Pakistani Muslims living in Norway, and they often face discrimination and exclusion from both the majority society and mainstream Muslims (Færseth, 2021; Naserah, 2022). Their narratives of persecution have shaped their image as “good Muslims” in the Western world, which has promoted their integration and positive visibility. Simultaneously, they emphasize the preservation of their culture, traditions, and religion through a close-knit community based on religious beliefs (Appleyard, 2015; Kelso, 2023).

### 3.2 Participants and recruitment

Participants were recruited from one of the largest cities in Norway in two phases in 2021 and 2022. The participants were recruited through snowball sampling with the assistance of key informants at a women's organization and via social media (Robinson, 2014). The 19 family caregivers included in the study were women between 25 and 62 years of age who provided unpaid care for older family members for at least six months. The study prioritized diversity among participants in terms of migration history, age, education level, marital status, family size, and generation (see Table 1).

**Table 1:** Characteristics of participants.

Characteristics of participants			
***1. Caregivers***		***4. When migrated to Norway***	
Daughter	12	Migrated in adulthood	9
Daughter-in-law	5	Migrated in childhood	5
Sister	1	Born in Norway	5
Granddaughter	1		
***2. Age (in years)***	***No. of Participants***	***5. Employment status***	***No. of Participants***
20–30	2	Homemaker	4
30–40	2	Part-time	4
40–50	7	Full-time	10
50–60	5	Unemployed	1
60–70	3		
***3. Marital status***		***6. Care model***	
Married	11	Co-residence	5
Unmarried	2	Rotational care (older adults take turns at children's houses)	5
Divorced	5	Rotational care (children stay nearby)	9
Widowed	1		

### 3.3 Semi-structured interviews

The first author conducted 18 semi-structured individual interviews (16 individual and 2 follow-up) and two group interviews with two participants each. The group interviews, involving siblings or relatives (sister and sister-in-law), were carried out to accommodate the participants’ preferences and offered insights into intra-generational dynamics in arranging care. Saturation was reached when new codes no longer added to the overall story. The questions in the interview guide focused on the following topics: organizing care; dividing responsibilities among family members; challenges faced by caregivers and their coping strategies; the impact of caregiving on work, family life, and relationships; and perceptions of providing sufficient care. Over half of the interviews (12) took place at a mosque, while the rest were held in a public library (4) or through a video call (4). In addition to conducting the interviews, the first author engaged in informal conversations with the women during mosque events to gain further insight into the Ahmadiyya community. Interviews were conducted in Urdu or English and were audio-recorded, transcribed, and translated verbatim into English. The interviews ranged from 45 min to 3 h in duration.

### 3.4 Data analysis

A reflexive thematic analysis guided by the work of Braun et al. (2022) was employed to analyze and generate themes in the dataset (see Table 2). The process involved six phases. First, the first author, who speaks Urdu and English, familiarized herself with the transcripts by reading and listening to the audio recordings several times and writing down immediate thoughts and ideas. In the second phase, the co-authors shared and co-generated codes from two reference transcripts. Subsequently, the first author coded the remaining transcripts and organized data around similar meanings, which was further shared with and discussed among the co-authors. In the third phase, codes were further organized into potential themes related to family caregivers’ experiences and conflicts in daily caregiving. In the fourth and fifth phases, these themes were refined through discussions among the co-authors to ensure comprehensive coverage of the data. The final phase involved producing the report (Braun et al., 2022).

**Table 2:** Example of the analysis process.

Meaning unit	Codes	Subthemes	Themes
− I do not know what will happen in the future – if we could take care of her if she cannot move by herself. If I were at home, there would be no issue, but I work, and the other [pointing to her sister] also does not have good health.	− Work issues and care − Health issues and care − Caregiving is difficult	− Uncertainty about future caregiving	− The need to share caring responsibilities with family
− I am relaxed now and staying here at the interview only because I know my daughter is at home taking care of my mother. Otherwise, my mother could not be left alone, and it would not be possible for me to come here today for the interview.	− Shared care − Divide care responsibilities − Lack of flexibility in time while caring alone	− Flexibility in time while sharing care	

The authors brought diverse perspectives to the data analysis due to their varied academic backgrounds (nursing, social and health sciences), research experiences with migrant families, ethnic backgrounds (Asian, African, and European) and genders. All authors contributed to constructing, defining, and redefining the themes through discussion until a consensus was reached, enhancing the study's rigor and trustworthiness.

### 3.5 Ethical considerations

The study was reported to the Norwegian Center for Research Data (NDS). NSD ensures that research is carried out in accordance with applicable data protection laws and regulations. This involves assessing how research data is collected, stored, processed, and shared, and ensuring that these processes respect the individual's right to privacy. All the participants were informed about the study and notified that they could withdraw from the study at any time without needing to state a reason. Written consent was obtained in the face-to-face interviews, whereas verbal consent was received in the video-call interviews. The anonymity of participants was maintained in all transcripts and in the presentation of the results.

## 4 Results

The analysis produced four themes that illustrate the dilemmas faced by caregivers in their daily lives, where the practical aspect of caregiving was not always aligned with the ideal context of care as a moral practice. The four themes are as follows: the need to share caregiving responsibilities with family, balancing personal relations in managing care, lack of privacy while caregiving, and feelings of inadequacy.

### 4.1 The need to share caregiving responsibilities with family

The participants identified various family care arrangements ranging from sole caregivers to family members sharing responsibilities. Co-residing with parents was common among unmarried caregivers but rare among married ones. Single parents typically moved in with one of their children or moved between their children's households, often at intervals of a few days, weeks, or months. Meanwhile, family caregivers who cared for both parents often lived a short drive from their parents’ house.

As they aged, older family members required more assistance with physical tasks and personal hygiene. Parents in difficult economic situations also needed help with travel, clothing, and food. All of the participants, especially those who were employed, expressed feelings of uncertainty about their capacity to continue caring for older relatives, and they were often unsure about the prospects of family care. A few caregivers whose older parents migrated later in life for family care i.e. zero-generation migrants (Nedelcu, 2023) reported their higher dependency due to language barriers and unfamiliarity with Norwegian daily life. This often led to diversion of their caregiving responsibilities from caring for their parents-in-law to now caring for their own parents (zero generation).

Additionally, the increasing age of some caregivers and their own health issues made their care responsibilities difficult to fulfill:

I do not know what will happen in the future – if we could take care of her if she cannot move by herself. If I were at home, there would be no issue, but I work, and the other [pointing to her sister] also does not have good health. (Participant 4, age 60–70, migrated to Norway in adulthood)

Sharing care responsibilities within the family was common and helped to alleviate the care burden for many participants. Because they no longer had to be constantly present at home to look after their older relatives, they had more time and flexibility:

I am relaxed now and staying here at the interview only because I know my daughter is at home taking care of my mother. Otherwise, my mother could not be left alone, and it would not be possible for me to come here today for the interview. (Participant 7, age 60–70, migrated to Norway in adulthood)

Although some caregivers voiced the need for their male siblings to contribute more, female siblings still took on a significantly larger share of care duties compared to males. Female caregivers provided more practical help with, for instance, household work, physical assistance, personal hygiene, and emotional support, whereas male siblings mainly checked on their parents through phone calls or visits, chatted with them, and provided financial support.

Due to the perceived tradition of gender segregation (*purdah*) in South Asian Islam and Ahmadi Islam (Gualtieri, 2004), there was a gendered limitation in providing care. The participants noted that it was rare for a son to assist with his mother's personal hygiene routine; thus, the pressure of caregiving remained on daughters (or daughters-in-law). Some participants expressed concerns about their aging fathers and fathers-in-law, who they worried may be more likely than older female relatives to end up in a nursing home once their health significantly declines:

Your daughter or daughter-in-law cannot do the work of men [maintaining personal hygiene]. There will be problems if they get very sick at home. I could not help how a man could help. (Participant 6, age 50–60, migrated to Norway in adulthood)

Though participants did not complain about providing care, they commented on the difficulty of maintaining the personal hygiene of older male relatives due to *purdah* as well as physical limitations due to, for example, men being taller and heavier than women.

The definition of care varied across participants. Some of them interpreted caregiving as providing both physical assistance as well as emotional support; being present for their older relatives. The later perspective was more common among some of the Norwegian-born participants.

Care does not mean that children need to change our diapers or that they need to move in with us, but it is about feeling that they are there for us as well. Having that feeling for your parents also could be defined as care, right? (Participant 13, age 40–50, born in Norway)

Many caregivers expressed a preference for home care services over nursing homes. In home care settings, nurses are expected to handle personal hygiene, while family members retain responsibility for other aspects of caregiving. However, despite their preference, very few of the caregivers actually used these services, and they seemed hesitant to openly discuss their use. While active caregivers perceived that they did not have a choice regarding their care responsibilities, they emphasized the importance of providing choices for younger generations. Those who viewed themselves as self-sufficient and economically independent believed that their own children might not feel as obliged to provide care for them as they felt for their parents.

### 4.2 Balancing personal relations in managing care

Throughout the interviews, the relational dynamics between caregivers, care receivers, and other family members, including children, husbands, siblings, and their partners, significantly influenced family care arrangements. Caregivers who were responsible for their in-laws were often in transnational cousin marriages, resulting in closer relationships, more active involvement in caregiving, including frequent visits to their in-laws’ homes, and better communication with their husbands’ siblings regarding the sharing of care responsibilities. Additionally, in cases of cousin marriages, not fulfilling their caregiving role could compromise not only their relationships with their in-laws but also their own parents’ standing within the family:

When I had conflicts with my husband and my in-laws [who were also her uncle and aunt], I used to hide these issues from my parents as much as possible. But, in the end, not only did I lose my relationship with my uncle and aunt: but my mother lost her relatives too. (Participant 12, age 40–50, born in Norway)

All five daughters-in-law in our study had transnational marriages and have lived in Norway for at least 20 years, with all participants being over 40 years old. While transnational marriages are prevalent in the Ahmadi community, our study highlighted the decreasing involvement of younger generation daughters-in-law in family care. One participant expressed the different care expectations among younger daughters-in-law with transnational marriages compared to older migrants and their descendants.

When parents want to bring daughter-in-law from Pakistan, they think that they know Pakistani culture and they are not Norwegians [laughs]. But Pakistan is not the same anymore. This is the reality here in Norway that our parents are very focused/invested that we must be in touch with our Pakistani culture, values, and religion since our childhood. However, when the daughters-in-law come from Pakistan, they do not come to Norway to serve their in-laws and take care of them. This is not the mentality. They come here because they want to work, become independent, and for freedom. That is why you don't find anyone who lives with their in-laws. (Participant 12, age 40–50, born in Norway)

A few participants who were born in Norway or migrated in childhood mentioned that caring for parents should be the responsibility of their children, not their daughters-in-law. A participant who was born and raised in Norway, where both her parents and parents-in-law also resided, felt responsible only for taking care of her own parents and expressed that caring for her parents-in-law was the responsibility of her husband and his siblings. In comparison, such flexibility in decision-making about caregiving was not common among daughters-in-law in transnational marriages (1st generation) who came to Norway in adulthood. Often, those with transnational marriages also expressed greater worries about straining their marital relationships while taking care of their own parents who had moved to Norway (either permanently or temporarily, i.e. zero generation).

While caring for my mother, I try hard to manage the house and our relationship [marital] smoothly. If I was doing it for his mother, and I was unable to manage household chores, he could have ignored it, but it is not the same for my mother. If it was for two or three days that she is living with us, then it would be fine, but if someone is living for a long time, it becomes difficult. (Participant 1, age 50–60, migrated to Norway in adulthood)

These worries about straining marital relationships influenced caregiving arrangements. To prevent long-term stays by parents in one residence, siblings needed to share the responsibility of caring for their parents through rotational care. Some participants suggested that caregiving might be easier for their unmarried sisters. Co-residence with their parents or a single parent was only common among daughters who were either unmarried or divorced. Furthermore, there was ongoing intra-gender negotiation among female caregivers regarding who should provide more care. Sisters or sisters-in-law with young children of their own contributed the least to caregiving for older adults, which resulted in some participants, with few siblings, becoming the sole caregiver:

My brother and his wife have two small children, so my sister-in-law cannot contribute to taking care of my father. So, he moved in with me. (Participant 5, age 50–60, migrated to Norway in childhood)

Many caregivers from the so-called sandwich generation (Boyczuk and Fletcher, 2016) found it difficult to balance their time, as they were primarily responsible for childcare in addition to eldercare. Both parties under their care at home – older adults and younger children – had particular routines, food preferences, activities, and lifestyles, and the participants acted as a bridge between them. Hence, the participants found that the process of balancing the two roles of caregiver and mother was a paradoxical situation: on the one hand, prioritizing caregiving for older relatives limited the participants’ time for their children, who felt neglected; on the other hand, prioritizing their role as a mother compromised the quality of care that they could provide for elders.

### 4.3 Lack of privacy while caregiving

Participants explained that families who lived together in multi-generational arrangements were perceived as having close-knit relationships and greater prestige within their community. However, regardless of whether they shared housing or lived nearby, caregivers expressed concerns about physical and private space. Some participants found that sharing housing could result in parents-in-law interfering in family decision-making, and they were therefore inclined to live separately. Some also stressed that many differences of opinion in a joint family can generate conflict between couples. Sharing housing with older adults also influenced the lifestyle of caregivers and their children, as they were careful not to offend the older adults.

For example, my daughter would like to go around and dress up differently, but she cannot, obviously, because she does not want to feel uncomfortable when my father is there and so am I. Also, if I wanted to have my colleagues from work visit me, I probably wouldn't invite the male, because I don't want to offend my dad. (Participant 5, age 50–60, migrated to Norway in childhood)

Many participants remarked on the importance of having a private space. Having a separate bedroom and attached bathroom for older family members afforded more physical privacy for both the older adults and the other family members. For some participants, the lack of a separate physical space for older family members created difficulties even in contributing to rotational care:

I think having the bed for my mother in the living room is problematic because, in the living room, a person feels like she is outside, and there is no privacy for her [mother]. Also, if you want to talk, a person [her nuclear family/husband and children] might have a problem because a person cannot share everything in front of others. (Participant 1, age 50–60, migrated to Norway in adulthood)

Co-residence compromised physical space, and caregivers had to restrict private conversations at home with their family, where the older adults were often not perceived as members of the nuclear family. Many participants who lived nearby stated that having a separate residence gave them more choices about when to provide care:

It is easier now. You can go to them and help. They are not too close or far away; there is enough distance. (Participant 10, age 40–50, migrated to Norway in adulthood)

In contrast, a few participants expressed that sharing housing kept them in a caregiving role 24/7 and burdened them with constant pressure to ensure their parents’ well-being while co-residing due to gender roles and certain expectations that parents have of their children.

### 4.4 Feelings of inadequacy

The feeling of being an inadequate caregiver and not being able to provide enough care was reported by several caregivers. Some were born in Norway, while others arrived in adulthood. Participants frequently compared themselves to their mothers, who were predominately housewives and were perceived as ideal caregivers in Pakistani context because they readily made sacrifices, prioritized others, and served the family. Participants with jobs expressed shame about not managing household work as perfectly as their mothers had.

When you come home exhausted after being at work all day. I know my father is used to staying in a clean place because my mother used to be at home all the time. Everything was shining, and she was the best cook ever, but I cannot do everything. I feel ashamed. (Participant 5, age 50–60, migrated to Norway in childhood)

Many participants, especially those with children, acknowledged the challenge of dedicating enough time to caregiving. Designating time for their children compromised their availability for their older parents, which evoked feelings of guilt about being unable to include parents in activities with children. Some participants reduced their participation in social activities to alleviate their guilt about leaving older family members behind.

However, discussions about the difficulties of managing caregiving rarely occurred within families, particularly between care receivers and caregivers, which contributed to the poor psychological well-being of family caregivers.

I cannot tell others, “Oh, I am tired.” So, you keep everything inside you, and due to that, subconsciously, I think it became a factor of stress in your life, and that frustration may be going out to your children, your partner or your health. (Participant 2, age 40–50, migrated to Norway in childhood)

Most participants feared disrespecting their older parents by vocalizing their challenges, but they experienced hopelessness, loneliness, and stress when suppressing their emotions. Some found mental peace in religion and prayer, which helped them shift their focus from the burden of caregiving responsibilities to the positive aspects of caregiving.

## 5 Discussion

This study has explored family caregiving arrangements for older migrants from the Ahmadiyya community in Norway. The participants reported that caregiving for older adults was primarily organized within the family, which is consistent with findings from other studies (Lillekroken et al., 2023; Shrestha et al., 2023). Although family members felt a shared sense of responsibility for eldercare, their experiences highlight the contradictions and dilemmas of caregiving and the resulting feelings of ambivalence; a simultaneous presence of solidarity and conflict regarding caring for one's older family members (Connidis, 2015; Lüscher and Pillemer, 1998; Park, 2014). Caregivers strived to navigate this ambivalence by adjusting the care arrangements and emphasizing the emotional aspects of care; still, many continued to feel guilty about their caregiving role. A few strived to adapt to innovative ways of caregiving, such as sharing the care responsibilities with formal care or home care services.

While participants acknowledged a growing desire to share eldercare responsibilities, they primarily did so within the family system through a rotational care arrangement. Detailed studies on rotational care practices are limited apart from a notable study from Denmark focusing on dementia care (Nielsen et al., 2021). Contrary to prevailing assumptions associating rotational care with more intensive caregiving, our findings indicate that this practice extends beyond dementia care to seemingly less intensive caregiving scenarios. Although sharing care responsibilities was deemed important, participants in transnational and/or cousin marriages who migrated in adulthood often coped through captivation by fulfilling their caregiving roles and responsibilities toward their in-laws rather reluctantly compared to those born and raised in Norway. In this study, ‘captivation’ refers to a continuous struggle with ambivalence, often inadequately expressed in words, leaving ambivalent feelings unacknowledged and care norms reluctantly maintained (Lüscher and Hoff, 2013; Park, 2014). Comparatively, participants in transnational and/or cousin marriages had fewer options for managing ambivalence and resisting normative caregiving obligations (Connidis and McMullin, 2002; Katz and Lowenstein, 2010).

Co-residence with older parents emerged as a prominent factor evoking IGA in parent-child relationships. While living together can symbolize a strong family and closer relationships (Aarset, 2020), it compromised the privacy and autonomy of the adult children and their families in our study. Younger generations in migrant-origin families tend to uphold traditional caregiving norms from their parents’ country of origin (Albert, 2023; Horn, 2023). At the same time, they are more likely than the older generation to adopt the receiving society's values of individualism, autonomy, and choice in family relationships (Gu, 2018; Aarset, 2020). This demonstrates that their values and norms are shaped by transnational social fields, comprising multiple interlocking networks of social relationships through which ideas, practices, and resources are unequally exchanged, organized, and transformed (Levitt and Schiller, 2004; Schiller et al., 1995). In previous studies, living separately from one's children was associated with the uncertainty of receiving informal care for older adults (Arora et al., 2020), and they felt ambivalent when their children decided to institutionalize them (Khan, 2023). However, in our study, such arrangements were beneficial for family caregivers in managing ambivalence. Not co-residing provided a certain distance that granted caregivers more autonomy in their caregiving decisions. Hence, living in a separate residence nearby was perceived as a way to reconcile and adapt intergenerational expectations and move toward atomization (without the agreement of parents) or emancipation (with the agreement of parents) by caregivers (Lüscher and Hoff, 2013). Hence atomization involves disengagement to avoid conflict and ambivalence, whereas emancipation entails openly acknowledging ambivalence, allowing individuals to accept both conflict and cooperation (Lüscher and Hoff, 2013; Park, 2014).

Despite the changes in co-residence norms, the expectation that eldercare would be given by the family persisted even when caregivers lived separately. The implementation of rotational care practices offered one approach to uphold traditional care norms, as seen in Pakistan, while accommodating the needs and preferences of family caregivers within the Norwegian context, in managing the care of older adults within the family system. However, rotational care was not an adequate solution when there were unequal intra-generational contributions to caregiving. The complexity of intra-generational relationships and their influence on intergenerational caregiving can result in collective ambivalence (Yao, 2023). In line with previous literature, our study found gendered disparities in caregiving duties, as women predominantly assumed the caregiving role (Liversage, 2023; Shrestha et al., 2023; Stenberg and Hjelm, 2023).

The challenges experienced by women in navigating their caregiving roles are intertwined with gendered expectations of caregiving, which introduces additional difficulties (Ahmad et al., 2022; Khan, 2023). Thus, gender plays a crucial role in shaping these experiences (Willson et al., 2003) and women who bear a larger caregiving burden are likely to experience ambivalence differently than men (Katz and Lowenstein, 2010; Yao, 2023). Gender, in combination with the transnational nature of marriage for some participants, also increased ambivalence toward caregiving due to fears of disrupting marital relationships. Participants with children also adopted captivation as a strategy to manage ambivalence (Lüscher and Hoff, 2013). Gendered expectations of care for both children and parents (Ahmad et al., 2022; Tavernier and Draulans, 2018) combined with women's participation in the labor market as a key indicator of how well migrants and their descendants are integrating into Norwegian society (Aarset, 2015, 2020), placed the caregivers in our study in conflicting social positions within family dynamics. This often generates significant multi-faceted ambivalence in intergenerational care.

In our study, the family caregivers’ gender and ethnicity appeared to influence their ambivalence toward their older relatives. The threat of facing social exclusion for not conforming to prevalent care norms is a prominent concern in migrant communities (Draulans and De Tavernier, 2020; Tavernier and Draulans, 2018), and our study group of the Ahmadiyya community was no exception. In this regard, limited agency due to social structures appeared to increase their IGA (Connidis and McMullin, 2002; Pillemer et al., 2012). As members of a small, close-knit community, the participants upheld more traditional eldercare norms (Appleyard, 2015; Kelso, 2023). The fear of social exclusion reinforced their adherence to traditional eldercare norms since deviating from them could be negatively viewed by the community and potentially result in atomization, which diminishes mutual cohesion and leads to conflicting intergenerational relationships (Lüscher and Hoff, 2013).

An intriguing finding of our study is that some family caregivers had the agency to negotiate the organization of family care according to their preferences, yet they were not in a position to change the traditional care norms that prioritize family as the primary source of eldercare. For example, while some family caregivers used innovative approaches of shared caregiving through rotational care practices or not co-residing with parents, such innovations do not contradict the traditional care norms of the family system – they merely adjust them. Hence, the use of captivation as a strategy to cope with IGA (Lüscher and Hoff, 2013; Park, 2014) was profound among family caregivers in our study. Through rotational care practices, care responsibilities for older adults remain within the family, but the care burden is distributed among family members. This approach not only provides respite care but also allows individuals to take short breaks from caregiving when other family members take over the care responsibilities (Nielsen et al., 2021; Zarzycki et al., 2023).

In the current study, female caregivers who were unmarried, divorced, widowed, childless, or aging themselves contributed disproportionately to caregiving compared to their female siblings who had young children, were married, or worked. High care obligations can result in increased stress and vulnerability (Nielsen et al., 2018) and may reduce the already-low labor market participation of migrant-background women in their fifties and sixties who have older parents (Syse and Tønnessen, 2022b). In our study, participants voiced concerns about the need for equal contributions from all siblings. Although their male siblings contributed economically, they often upheld gendered caregiving norms, which was another key source of ambivalence for family caregivers. Another study in Norway among the majority population has demonstrated that daughters were more likely to enter a caregiving role, especially in families where the daughter was the only female sibling (Vergauwen and Mortelmans, 2021).

We also observed an intra-gender shifting of care responsibilities in our study, as caregiving responsibilities were increasingly ascribed to daughters instead of daughters-in-law. Compared to daughters-in-law who were born in Pakistan, those who were born in Norway had more negotiating power to prioritize caring for their mothers rather than their parents-in-law. Consistent with our research, a study among Turkish migrants in Denmark has highlighted the agency of Danish-born daughters-in-law in resisting caregiving expectations for their in-laws and shifting them to their husbands and husbands’ siblings (Liversage, 2023). Similarly, younger daughters-in-law with transnational marriages were perceived to have significant agency in resisting caregiving expectations in our study. This highlights how migrants may freeze their care practices and norms over time, even as their country of origin evolves (Rytter, 2013; Aarset, 2015). Thus, marriage migrants do not always guarantee additional family members contributing to family care. This contrasts with findings from a study in Denmark, which indicated that older immigrants received better family support when children-in-law arrived as marriage migrants (Liversage, 2017). Notably, while the caregiving responsibilities shifted from daughters-in-law to daughters, the gendered imbalances in caregiving did not decrease, as the caregiving responsibilities merely shifted to other relatives of the same gender.

Our findings indicate that family caregiving burden, increasing privacy concerns and relational dynamics with husbands, as well as the popularity of nuclear family structures could lead to either older migrants rotating between their children's houses at short intervals or children visiting their parents’ homes less frequently at longer intervals. Despite the shared care responsibilities among family members, each person's limited ability to contribute due to their individual circumstances further underscores the precarious situation of sole caregivers. Both sole caregivers and caregivers who shared responsibilities expressed uncertainty about the future, when older individuals would develop more intensive care needs. While family caregiving is associated with personal growth, self-efficacy, satisfaction, a sense of gratification, and closer relationships with care receivers (Zarzycki et al., 2023), ambivalent settings have been found to worsen depressive symptoms, psychological well-being, and quality of life (Yao, 2023) and decrease the quality of relationships with both care receivers and other family members (Lüscher and Hoff, 2013). Intergenerational ambivalence between an adult child and their aging parent can influence the present and future quality of care (Lüscher and Hoff, 2013), as family caregivers who experience high ambivalence are more likely to discontinue care (Pillemer et al., 2019).

The ambivalence (Connidis, 2015) experienced at the individual level by caregivers in our study is reflected in their feelings of inadequacy and resonates with the broader normative perceptions of caregiving within their ethnic community. Individuals inspired by collectivistic norms prioritize family relationships and derive their sense of identity from relationship and group membership (Nielsen et al., 2018). In our study, caregivers often grappled with simultaneous contradictory attitudes about the quality of care they provided, with traditional qualities (e.g., selflessness, tolerance, patience, sacrifice) defining the notion of a “good” caregiver. Obediently caring for one's parents without complaining has often been equated with the expression of gratitude toward them (Ismail, 2021). In our study, many women found themselves in a transitional phase, as their circumstances and caregiving approaches were changing, and they had higher education levels and labor market participation than their mothers and mothers-in-law. Nonetheless, normative perceptions of caregiving remained deeply rooted and caused them to perceive themselves as falling short of the expectations associated with a “good” caregiver in their community. This phenomenon shows how IGA is greater in women due to gendered expectations around family labor (Connidis and McMullin, 2002; Willson et al., 2003). On the other hand, it also indicates that older migrants may not be receiving adequate care, as the higher perceived caregiving obligations do not necessarily translate into higher levels of actual care (Horn, 2023).

Among both sole caregivers and those sharing care in our study, there were widespread feelings of guilt about not being an adequate caregiver and not doing enough for parents. Similar findings have been reported by Khan (2023) in a study of South Asian Muslim families in the US. The gendered nature of caregiving has resulted in the image of an “ideal” caregiver as someone who either enjoys caregiving (Connidis and McMullin, 2002; Willson et al., 2003) or makes sacrifices and suppresses their true feelings (Gu, 2018). Feelings of ambivalence deviate from this ideal image and create a situation in which captivation is the only coping strategy. Furthermore, the reluctance to express these ambivalent experiences hinders communication and sharing and prevents caregivers from seeking help from informal or formal sources. The findings of this study highlight a shift from instrumental to emotional care that reflects the dynamic definition of family care. Although women's emotional involvement in caregiving is associated with a higher caregiving burden (Zygouri et al., 2021), it has also been linked to reduced ambivalence among older adults, particularly mothers (Guo et al., 2018). While families remain responsible for emotional support, it is noteworthy that participants showed a growing openness to utilizing formal services that provide instrumental and gender-matched eldercare.

### 5.1 Strengths and limitations

Qualitative measures are perceived to add depth to studies exploring ambivalence (Connidis, 2015; Lüscher and Hoff, 2013). The explorative, qualitative nature of our study and the semi-structured character of the interviews granted family caregivers the space and time to voice their experiences of caring for their older parents. Our approach produced rich data and sufficient transcripts to conduct an in-depth exploration of the ambivalence felt by family caregivers. Conducting the interviews in both Urdu and English allowed the participants to comfortably recall their experiences in their preferred languages.

Nevertheless, it is important to acknowledge some limitations of our study. First, we focused on interviewing adult children who provided care to their parents, and care arrangements for older adults without children were beyond the scope of the study. Second, we did not explore care receivers’ perspectives regarding their care arrangements, which could provide valuable insights to compare with their children's views on the caregiving arrangements. Finally, our study did not include any male family caregivers, whose experiences might have added further nuance to the gendered aspect of ambivalence.

### 5.2 Future implications

A better understanding of eldercare arrangements within migrant-origin families could inform more inclusive policies that are responsive to the needs of family caregivers. In addition, it could support health personnel in developing their competency to address diverse care circumstances and facilitating better interactions and collaboration. Understanding the circumstances of family care could be a catalyst for support networks in the community to help family caregivers navigate the complexities of caregiving by better managing the care burden and intergenerational relationships.

## 6 Conclusion

Caregiving for older adults is primarily arranged within the family, and women assume a larger share of caregiving responsibilities than men. Our findings highlight several shifts in eldercare arrangements from co-residence to rotational care and from instrumental to emotional care. Although participants recognized that rotational care practices are helpful for ensuring privacy, sharing care responsibilities, and balancing the multiple roles of female family caregivers, it is evident that relying solely on family care arrangements may not be sufficient. Such care arrangements often led to greater ambivalence among family caregivers and doubts about the ability to continue caring for older relatives in the future. These findings highlight the urgent need to establish modes of collaboration with formal care systems to ensure the support and well-being of both older adults and their family caregivers. Future research should explore family caregivers’ perspectives of formal care to address uncertainties surrounding the care of older migrants.

## Research ethics and participant consent

This study was approved by the Norwegian Center for Research (Reference number 271584). Written informed consent was obtained in the case of face-to-face interviews, whereas for Microsoft Teams interviews, verbal informed consent was received.

## CRediT authorship contribution statement

**Sunita Shrestha:** Writing – original draft, Methodology, Formal analysis, Data curation, Conceptualization. **Alistair Hunter:** Writing – review & editing, Supervision. **Jonas Debesay:** Writing – review & editing, Conceptualization. **Sanjana Arora:** Writing – review & editing, Supervision.

## Declaration of competing interest

The authors declared no potential conflicts of interest concerning the research, authorship, and/or publication of this article.

## Data Availability

The data supporting this study's findings are available on request from the corresponding author. The data are not publicly available due to privacy or ethical concerns.

## References

[bib0001] Aarset M.F. (2015). https://www.sv.uio.no/sai/forskning/aktuelt/arrangementer/disputaser/2015/summary.pdf.

[bib0002] Aarset M.F. (2020). Å holde hverdagslivet sammen». Hjem, familie og tilhørighet blant etterkommere av innvandrere fra India og Pakistan [Holding everyday life together». Home, family and belonging among descendants of immigrants from India and Pakistan]. Nor. antropol. tidsskr..

[bib0003] Ahmad M., van den Broeke J., Saharso S., Tonkens E. (2022). Dementia care-sharing and migration: an intersectional exploration of family carers' experiences. J. Aging Stud..

[bib0004] Albert I, torres S., Hunter A. (2023). Handbook On Migration and Ageing.

[bib0006] Appleyard, R.D. (2015). *Defining Community and Expressing Identity: A case Study of the Transplantation, Development and Adaption of the Ahmadiyya Jama'at in Kristiansand.* Monograph, University of Agder. https://uia.brage.unit.no/uia-xmlui/handle/11250/302028.

[bib0005] Albertini M., Mantovani D., Gasperoni G. (2019). Intergenerational relations among immigrants in Europe: the role of ethnic differences, migration and acculturation. J. Ethn. Migr. Stud..

[bib0007] Arora S., Straiton M., Bergland A., Rechel B., Debesay J. (2020). Renegotiating formal and informal care while ageing abroad: older Pakistani women's healthcare access, preferences and expectations in Norway. J. Migr. Health.

[bib0008] Berry J.W. (2005). Acculturation: living successfully in two cultures. Int. J. Intercult. Rel..

[bib0009] Boyczuk A.M., Fletcher P.C. (2016). The ebbs and flows: stresses of sandwich generation caregivers. J. Adult. Dev..

[bib63] Braun V., Clarke V., Braun V. (2022). Thematic Analysis : a Practical Guide.

[bib0010] Connidis I.A. (2015). Exploring ambivalence in family ties: progress and prospects. J. Marriage Fam..

[bib0011] Connidis I.A., McMullin J.A. (2002). Sociological ambivalence and family ties: a critical perspective. J. Marriage Fam..

[bib0012] de Valk H.A.G., Bordone V. (2019). Co-residence of adult children with their parents: differences by migration background explored and explained. J. Ethn. Migr. Stud..

[bib0013] Debesay J., Nortvedt L., Langhammer B. (2022). Social inequalities and health among older immigrant women in the Nordic countries: an integrative review. SAGe Open. Nurs..

[bib0014] Draulans, V., & De Tavernier, W. (2020). Shifts in intergenerational solidarity: eldercare in the Turkish community of a Belgian city. In I. V. Hoyweghen, V. Pulignano, & G. Meyers (Eds.), *Shifting Solidarities: Trends and Developments in European Societies* (pp. 201–227). 10.1007/978-3-030-44062-6.

[bib0015] Færseth J. Ahmadiyya-imam går ut mot samfunnsdebattant: – Sprer konspirasjonsteorier om forfulgt minoritet [Ahmadiyya imam goes out against social debater: - Spreads conspiracy theories about persecuted minority]. *Utrop; Norges første flerkulturelle Avis*. https://www.utrop.no/nyheter/nytt/243107/.

[bib0016] Gu C.J. (2018). Gender morality and emotion work in Taiwanese immigrant in-law relations. Gend. Place Cult..

[bib64] Gualtieri A.R. (2004). *The Ahmadis: Community, Gender, and Politics in a Muslim Society*.

[bib0017] Gulestø R., Lillekroken D., Halvorsrud L., Bjørge H. (2023). Different senses of one's place: exploring social adjustment to home-based care services among family caregivers from minority ethnic backgrounds who have relatives living with dementia. Dementia.

[bib0018] Guo M., Liu J., Xu L., Mao W., Chi I. (2018). Intergenerational relationships and psychological well-being of Chinese older adults with migrant children: does internal or international migration make a difference?. J. Fam. Issues..

[bib0019] Horn, V. (2023). Older migrants and care recipiency. In S. Torres & A. Hunter (Eds.), *Handbook On Migration and Ageing* (pp. 322–332). 10.4337/9781839106774.00039.

[bib0020] Høydal H.F., Wichstad E., Andreassen T. Norsk borger drept i Pakistan: − Vi kan nesten ikke tro det [Norwegian citizen killed in Pakistan:- We can hardly believe it]. *VG*. https://www.vg.no/nyheter/utenriks/i/wAn17G/norsk-borger-drept-i-pakistan-vi-kan-nesten-ikke-tro-det.

[bib0021] Ingebretsen, R., Thorsen, K., & Myrvang, V. (2015). *Livsmot og mismot blant aldrende kvinner med innvandrerbakgrunn:“Møteplasser er viktig! Det er kjempeviktig!”[Courage and discourage amongst older women with a migrant background:“It is really important to have a place to meet!”]*. Tønsberg: Forlaget Aldring og helse.

[bib0022] Ismail A.M. (2021). Care in practice: negotiations regarding care for the elderly in multigenerational Arab Muslim families in Denmark. Contemp. Islam.

[bib0023] Jacobsen, B.A., Larsson, G., & Sorgenfrei, S. (2015). The Ahmadiyya mission to the Nordic countries. In J. R. Lewis & I. B. Tøllefsen (Eds.), *Handbook of Nordic New Religions* (Vol. 11, pp. 359–373). Brill Handbooks on Contemporary Religion. 10.1163/9789004292468_023.

[bib0024] John W.C., Creswell J.D. (2023).

[bib0025] Katz R., Lowenstein A., Izuhara M. (2010). Ageing and Intergenerational Relations: Family recirpocity from a Global Perspective.

[bib0026] Kelso E. (2023). Truth in progress: second-generation Ahmadi-Muslim women performing integration in Germany. Südasien-Chronik - South Asia Chronicle.

[bib0027] Khan M.M. (2023). Of duty and diaspora: (*Re*)negotiating the intergenerational contract in South Asian Muslim families. J. Aging Stud..

[bib0028] Levitt P., Schiller N.G. (2004). Conceptualizing Simultaneity: a Transnational Social Field Perspective on Society. Int. Migr. Rev..

[bib0029] Lillekroken D., Halvorsrud L., Gulestø R., Bjørge H. (2023). Family caregivers’ experiences of providing care for family members from minority ethnic groups living with dementia: a qualitative systematic review. J. Clin. Nurs..

[bib0030] Liversage A. (2017). Twice as many helpers: unpacking the connection between marriage migration and older labour immigrants’ access to family support. Migr. Lett..

[bib0031] Liversage A. (2023). Care arrangements between family and state – developing hybrid scripts of ageing in a context of migration. Ageing Soc..

[bib0032] Lüscher, K., & Hoff, A. (2013). Intergenerational ambivalence: beyond solidarity and conflict. In *Intergenerational Relations: European Perspectives in Family and Society* (Vol. 39, pp. 39–64). Policy Press Scholarship Online. 10.1332/policypress/9781447300984.003.0004.

[bib0033] Lüscher K., Pillemer K. (1998). Intergenerational ambivalence: a new approach to the study of parent-child relations in later life. J. Marriage Fam..

[bib0034] Naserah Y. Norskpakistaneres behandling av Ahmadiyya-muslimer i Norge er en skam [Norwegian Pakistanis’ treatment of Ahmadiyya Muslims in Norway is a shame]. *Utrop; Norges første flerkulturelle Avis*. https://www.utrop.no/plenum/ytringer/308161/.

[bib0035] Nedelcu, M. (2023). Family reunification migrants and the zero generation. In S. Torres & A. Hunter (Eds.), *Handbook On Migration and Ageing* (pp. 196). 10.4337/9781839106774.00026.

[bib0036] Nielsen D.S., Minet L., Zeraig L., Rasmussen D.N., Sodemann M. (2018). Caught in a generation gap”: a generation perspective on refugees getting old in Denmark— a qualitative study. J. Transcult. Nurs..

[bib0037] Nielsen R.T., Waldemar G., Nielsen D.S. (2021). Rotational care practices in minority ethnic families managing dementia: a qualitative study. Dementia.

[bib0038] Northwood M., Ploeg J., Markle-Reid M., Sherifali D. (2018). Integrative review of the social determinants of health in older adults with multimorbidity. J. Adv. Nurs..

[bib0039] Næss A., Vabø M. (2014). Negotiating narratives of elderly care: the case of Pakistani migration to Norway. Ageing Int..

[bib0040] Park S.-M. (2014). Theory of intergenerational ambivalence: is it the perfect new lens for studying intergenerational relationships?. J. Popul. Ageing.

[bib0041] Pillemer K., Munsch C.L., Fuller-Rowell T., Riffin C., Suitor J.J. (2012). Ambivalence toward adult children: differences between mothers and fathers. J. Marriage Fam..

[bib0042] Pillemer K., Suitor J.J., Baltar A.L. (2019). Ambivalence, families and care. Int. J. Care Caring.

[bib0043] Robinson O.C. (2014). Sampling in interview-based qualitative research: a theoretical and practical guide. Qual. Res. Psychol..

[bib0044] Rytter M. (2013).

[bib0045] Sagbakken M., Spilker R.S., Ingebretsen R. (2018). Dementia and migration: family care patterns merging with public care services. Qual. Health Res..

[bib0046] Schiller N.G., Basch L., Blanc C.S. (1995). From immigrant to transmigrant: theorizing transnational migration. Anthropol. Q..

[bib0047] Schulz R., Beach S.R., Czaja S.J., Martire L.M., Monin J.K. (2020). Family caregiving for older adults. Annu. Rev. Psychol..

[bib0048] Schwartz S.J., Unger J.B., Zamboanga B.L., Szapocznik J. (2010). Rethinking the concept of acculturation: implications for theory and research. Ame. Psychol..

[bib0049] Shrestha S., Arora S., Hunter A., Debesay J. (2023). Changing dynamics of caregiving: a meta-ethnography study of informal caregivers’ experiences with older immigrant family members in Europe. BMC Health Serv. Res..

[bib0050] SSB. (2024). *Immigrants and Norwegian-born to immigrant parents*. Statistics Norway https://www.ssb.no/en/statbank/table/05183/tableViewLayout1/.

[bib0051] Stenberg J., Hjelm K. (2023). Migrant informal caregiver perceptions and experiences of caring for a family member with dementia: a systematic review and thematic synthesis. J. Clin. Nurs..

[bib0052] Sun, K.C.-Y. (2023). Intergenerational relations. In S. torres & A. Hunter (Eds.), *Handbook On Migration and Ageing* (pp. 25–34). Edward Elgar Publishing. 10.4337/9781839106774.00009.

[bib0053] Syse A., Tønnessen M. (2022). Flere eldre innvandrere blant framtidens brukere av omsorgstjenester. Tidsskrift for omsorgsforskning.

[bib0054] Syse A., Tønnessen M. (2022). Flere eldre innvandrere blant framtidens brukere av omsorgstjenester. Tidsskrift for omsorgsforskning.

[bib0055] Tavernier W.D., Draulans V. (2018). Negotiating informal elder care, migration and exclusion: the case of a Turkish immigrant community in Belgium. Int. J. Ageing Later Life.

[bib0056] Vangen H., Herlofson K. (2023). Why care? How filial responsibility norms and relationship quality matter for subsequent provision of care to ageing parents. Ageing Soc..

[bib0057] Vergauwen J., Mortelmans D. (2021). An integrative analysis of sibling influences on adult children's care-giving for parents. Ageing Soc..

[bib0058] WHO. (2018). *Health of older refugees and migrants* (978 92 890 5373 0). (Technical guidance on refugee and migrant health, Issue. https://www.euro.who.int/__data/assets/pdf_file/0003/386562/elderly-eng.pdf.

[bib0059] Willson A.E., Shuey K.M., Elder Jr G.H. (2003). Ambivalence in the relationship of adult children to aging parents and in-laws. J. Marriage Fam..

[bib0060] Yao, H. (2023). Ambivalence in family life during the era of falling fertility and population aging: theoretical and clinical considerations. In S. Chen (Ed.), *Social Work, Mental Health, and Public Policy in Diverse Contexts: Chinese and Cross-Cultural Perspectives* (pp. 201–218). Springer. 10.1007/978-3-031-36312-2_13.

[bib0061] Zarzycki M., Seddon D., Bei E., Morrison V. (2023). Why do they care? A qualitative systematic review and meta-synthesis of personal and relational motivations for providing informal care. Health Psychol. Rev..

[bib0062] Zygouri I., Cowdell F., Ploumis A., Gouva M., Mantzoukas S. (2021). Gendered experiences of providing informal care for older people: a systematic review and thematic synthesis. BMC Health Serv. Res..

